# Neonatal Mortality and Long-Term Outcome of Infants Born between 27 and 32 Weeks of Gestational Age in Breech Presentation: The EPIPAGE Cohort Study

**DOI:** 10.1371/journal.pone.0145768

**Published:** 2016-01-08

**Authors:** Elie Azria, Gilles Kayem, Bruno Langer, Laetitia Marchand-Martin, Stephane Marret, Jeanne Fresson, Véronique Pierrat, Catherine Arnaud, François Goffinet, Monique Kaminski, Pierre-Yves Ancel, EPIPAGE study group

**Affiliations:** 1 INSERM, U-1153, Epidemiology and Biostatistics Sorbonne Paris Cité Center, Obstetrical, Perinatal and Pediatric Epidemiology Team, DHU Risk in Pregnancy, Paris, France; 2 Department of Obstetrics, Groupe Hospitalier Paris Saint Joseph, Paris Descartes University, Paris, France; 3 Department of Obstetrics and Gynecology, Hôpital Trousseau, Assistance Publique des Hôpitaux de Paris, University Pierre et Marie Curie, Paris, France; 4 Department of Gynecology Obstetrics, Strasbourg University Hospitals, Hôpital de Hautepierre, Strasbourg, France; 5 Department of Neonatal Medicine, Rouen University Hospital, Rouen, France; 6 INSERM, AVENIR Research Group & Department of Neonatal Medicine and Intensive Care and Regional Center for Diagnosis and Research on Developmental Language and Behavioural Disorders, Rouen Institute for Biomedical Research, Rouen, France; 7 Medical Information Department, Regional Maternity University Hospital, Nancy, France; 8 Department of Neonatal Medicine, Hôpital Jeanne de Flandre, Lille, France; 9 INSERM, UMR 1027 Inserm, Toulouse III University, F-31000, Toulouse, France; 10 Clinical epidemiology unit, University Hospital, F-31000, Toulouse, France; 11 Department of Obstetrics and Gynecology, Port-Royal Maternity, Groupe Hospitalier Cochin-Broca-Hôtel Dieu, Assistance Publique des Hôpitaux de Paris, Paris, France; Hôpital Robert Debré, FRANCE

## Abstract

**Objective:**

To determine whether breech presentation is an independent risk factor for neonatal morbidity, mortality, or long-term neurologic morbidity in very preterm infants.

**Design:**

Prospective population-based cohort.

**Population:**

Singletons infants without congenital malformations born from 27 to 32 completed weeks of gestation enrolled in France in 1997 in the EPIPAGE cohort.

**Methods:**

The neonatal and long-term follow-up outcomes of preterm infants were compared between those in breech presentation and those in vertex presentation. The relation of fetal presentation with neonatal mortality and neurodevelopmental outcomes was assessed using multiple logistic regression models.

**Results:**

Among the 1518 infants alive at onset of labor included in this analysis (351 in breech presentation), 1392 were alive at discharge. Among those eligible to follow up and alive at 8 years, follow-up data were available for 1188 children. Neonatal mortality was significantly higher among breech than vertex infants (10.8% vs. 7.5%, P = 0.05). However the differences were not significant after controlling for potential confounders. Neonatal morbidity did not differ significantly according to fetal presentation. Severe cerebral palsy was less frequent in the group born in breech compared to vertex presentation but there was no difference after adjustment. There was no difference according to fetal presentation in cognitive deficiencies/learning disabilities or overall deficiencies.

**Conclusion:**

Our data suggest that breech presentation is not an independent risk factor for neonatal mortality or long-term neurologic deficiencies among very preterm infants.

## Introduction

Poorer outcomes have been reported for term infants in breech compared with vertex presentation [1–3]. These may result either from underlying conditions that might cause breech presentation, such as congenital anomalies [4] or intrauterine growth restriction, or from perinatal complications during labor[5] and delivery [6]. Some authors [3,7], but not all [8,9] consider that breech presentation is an independent risk factor for neonatal morbidity and mortality for term newborns.

The question of outcome according to presentation at birth also arises for preterm infants. Preterm birth is associated with a higher prevalence of breech presentation. The frequency of breech deliveries decreases as gestational age increases and affects approximately 3–4% of all term infants. Scheer and Nubar reported a prevalence of breech presentation ranging from 9% at 33 to 36 weeks gestation to 28% at 25 to 28 weeks [10].

It has been suggested that neonatal mortality in preterm infants might be independently associated with breech presentation [11,12]. This idea, however, is based on studies subject to bias and relies on old data that might not reflect current clinical practices. To our knowledge, no published study has compared the long-term morbidity, especially neurocognitive deficiencies, of preterm infants according to their presentation—breech or vertex. Such a study would require a large sample size, specific developmental assessments, and long-term follow up. It is therefore not known if breech presentation is really an independent risk factor in preterm infants for neonatal mortality and for short- and long-term morbidity or if the suggested increased risk is due to other factors, such as the underlying condition of the fetus or the mode of delivery [13].

The purpose of this study is to compare the outcomes of very preterm infants according to whether they were born in breech or vertex presentation and thereby determine whether breech presentation is an independent risk factor for neonatal mortality and morbidity and for long-term neurologic morbidity in this population. For this study, we used the data of a large prospective cohort of very preterm infants born in 1997 and followed up to the age of 8 years.

## Methods

### Population study

The EPIPAGE (Etude EPIdémiologique sur les Petits Ages Gestationnels, epidemiologic study of early gestation ages) study is a population-based prospective cohort study that included all births occurring from 22 to 32 completed weeks of gestation in 1997 in 9 French regions. It has been described in detail elsewhere [14]. At recruitment in the maternity or neonatal unit, parents were told about the study and given written information. Verbal consent was provided to the local teams in charge of the study. The study was approved by the French Commission Nationale de l'Informatique et des Libertés (the French data protection agency).

Although all infants born before 33 weeks were enrolled in the EPIPAGE cohort, we chose to restrict this analysis to those born at 27–32 weeks in order to avoid the wide variability of obstetric and neonatal care practices before 27 weeks [15,16]. This study was also restricted to singletons and to infants alive at onset of labor or before delivery when cesarean delivery was decided before labor. Moreover, infants were excluded if they had a congenital malformation identified as severe independently by two obstetricians (EA and GK) or were born in a nonvertex nonbreech presentation, or had missing data for presentation.

### Maternal and perinatal characteristics

Neonatal and obstetric data were extracted from medical records, and social characteristics were collected by maternal interview. Socioeconomic status (SES) was recorded according to the French classification of occupations and social position and grouped into five categories. Family SES was defined as the highest of the mother or father’s occupation, or if she lived alone, the mother’s occupation. Maternal age, country of birth, and education level were also recorded.

Medical information included previous and current obstetric history. Fetal presentation, antenatal administration of corticosteroids, mode of delivery grouped in 3 categories (vaginal birth, cesarean section during labor, cesarean section before labor), gestational age at delivery, and infant sex were considered. The obstetric complications leading to the preterm birth were recorded and grouped in 4 categories: 1- maternal hypertension or small-for-gestational-age fetus (SGA), defined as birth weight ≤10^th^ percentile (EPIPAGE internal reference) [17]; 2- spontaneous preterm labor or PPROM (preterm premature rupture of the membranes); 3- placenta praevia, abruptio placentae, or other hemorrhage; 4- other. The level of maternity unit facilities was recorded. In 1997 in France there were no national guidelines for the management of preterm infant in breech presentation delivery.

### Neonatal outcomes

Neonatal outcomes included death (including intrapartum death, death in the maternity unit or in the neonatal unit), called "neonatal death" in the text and tables, early-onset neonatal infection (confirmed infection of maternal origin), and late-onset neonatal infections (postnatally acquired infection treated with antibiotics for at least 7 days) as defined by Mitha et al [18], necrotizing enterocolitis, bronchopulmonary dysplasia (O2 dependency at 36 weeks), and neonatal cerebral lesions on neonatal cranial ultrasound, classified as major (cystic periventricular leukomalacia or intraparenchymal hemorrhage), moderate (intraventricular hemorrhage with primary ventricular dilatation (IVH) or ventricular dilatation or persistent echodensity), minor (subependymal hemorrhage or IVH without ventricular dilatation), or no lesions.

### Neurodevelopmental outcomes

The main steps of the follow-up included: 1) a standardized medical examination completed by the child’s treating physician at 2 years of age; 2) a standardised medical examination, including a short version of the Touwen neurologic examination [19] and a developmental assessment with the Kaufman Assessment Battery for Children (K- ABC) [20], at 5 years of age, performed by trained examiners in special centers set up for the study [14,21]; data on special care were also collected; 3) a postal questionnaire, sent to parents to investigate the child’s health and school situation at 8 years of age. Moreover, in 5 regions, a questionnaire was completed with the local office for people with disabilities (Maison Départementale des Personnes Handicapées) in 5 regions, to collect information on deficiencies, special schooling, and special care. All available information from all follow-up points was used to identify children with neuromotor, cognitive, neurosensory, or psychiatric deficiencies. Priority in grading severity was given to the most recent information. The methods and detailed classifications have been described in detail elsewhere [22]. Table 1 shows the main categories of deficiencies studied, ie neuromotor and cognitive.

**Table 1:** Classification of deficiencies. ^(1)^ [22]

**Neuromotor deficiencies**	
Severe CP	CP, unable to walk or walking only with aid at 8 or 5 years, or 2 years if no further follow-up.
Moderate CP	CP, walking without aid at 8 or 5 years, or 2 years if no further follow-up.
No CP, other motor disorder	No CP but MND2 at Touwen examination ^(2)^ at age 5 years
	or dyspraxia or motor coordination disorder (ICD F82, R26, R27) at 8 or 5 years, or 2 years if no further follow-up.
None identified	No CP and no other motor disorder identified ^(3)^.
**Cognitive deficiencies/learning disabilities**	
Severe	Mental retardation at 8 or 5 years (ICD F70-F79)
	or special school/class ^(4)^ at 8 years with MPC at 5 years <70 ^(5)^
	or no information at 8 years but MPC at 5 years <70
	or mental retardation at 2 years, if no further follow-up.
Moderate	Moderate/mild cognitive deficiency mentioned in MDPH^(6)^ record with no other details,
	or if in a mainstream class at 8 but having repeated one grade and/or receiving/needing special support at school ^(4)^
	or no information at 8 years, MPC between 70 and 84 at 5 years.
None identified	Mainstream class appropriate for age without any special support at 8 years
	or if no information at 8 years, MPC>85 at 5 years
	or if only medical examination at 2 or 5 years, no cognitive deficiency mentioned.
**Overall deficiencies**	
Severe	At least one of: severe CP, severe cognitive deficiency/learning disabilities,
	severe psychiatric disorder,^(7)^ epilepsy, visual deficiency or hearing deficiency.
Moderate	At least one of: moderate CP, other motor disorder, moderate cognitive deficiency
	or moderate psychiatric disorder^(8)^.
None identified	None of the above.

^(1)^ For each deficiency, the classification follows a priority order according to severity at the most recent step of the follow-up available.

^(2)^ Having Moderate Neuromotor Dysfunction (MND-2) at the short version of the Touwen neurological examination at the age of 5 years.^19^

^(3)^ Including children without CP or other neuromotor disorders but who had not been assessed with the Touwen examination.

^(4)^ Except for visual or hearing deficiency only. Visual deficiency: blindness (uni-or bilateral) or Rossano test ≤2 in both eyes at 5 years; Hearing deficiency: deafness in one or both ears or use of hearing aid at any age.

^(5)^ MPC = Mental Processing Composite of the Kaufman Assessment Battery for Children.^20^

^(6)^ Maison Départementale des Personnes Handicapées (local office for people with disabilities).

^(7)^ Autism, pervasive development disorders (ICD F84) at 8 or 5 years.

^(8)^ Hyperactivity or attention deficit disorder (ICD F90) or conduct disorder (ICD F91) as a reason for a visit to a psychiatrist or a psychologist at 8 or 5 years.

As explained previously in the Marret et al. paper [22], to reduce bias due to loss to follow-up and to be able to classify as many children as possible, we used all available data from all stages of follow-up (2, 5, and 8 years) for each deficiency to determine if it affected the child and how severely. Moreover, when information in one domain of development was missing at one stage of follow up, the child was considered free from this deficiency at that stage.

### Statistical analysis

Children in breech presentation at birth were compared to those in vertex presentation for the obstetric complication leading to the birth, mortality, neonatal morbidity, and long-term neurodevelopmental outcomes.

The comparisons were performed first for the entire study sample of preterm infants and then for the subset of births following spontaneous preterm labor or PPROM. Because complications leading to very preterm delivery can influence neonatal and long-term outcomes [23,24], we chose to study specifically the subgroup of spontaneous preterm births and PPROM. This context of birth is both the most frequent situation faced by obstetricians and a more homogenous group. All comparisons used the chi-square test.

Multivariate analyses using multiple logistic regression models were conducted to estimate the relation of fetal presentation to neonatal mortality and to neurodevelopmental outcomes. Adjusted ORs (adjORs) and 95% confidence intervals (CIs) were calculated adjusting first for gestational age (the main risk factor), sex, and antenatal corticosteroid use (also known risk factors for neonatal mortality and morbidity) [14,25], then adding SGA and PPROM, and thirdly, maternity unit level and mode of delivery, which are also suggested risk factors.[19,26] Because of the influence of social environment on cognitive development, analyses for cognitive deficiencies were further adjusted for parental SES, maternal age at delivery, and maternal country of birth. Statistical analyses were performed with SAS (SAS Institute Inc., Cary, NC), version 9.3. All tests were two-sided, and the level of significance was *P*<0.05.

## Results

**Fig 1:**
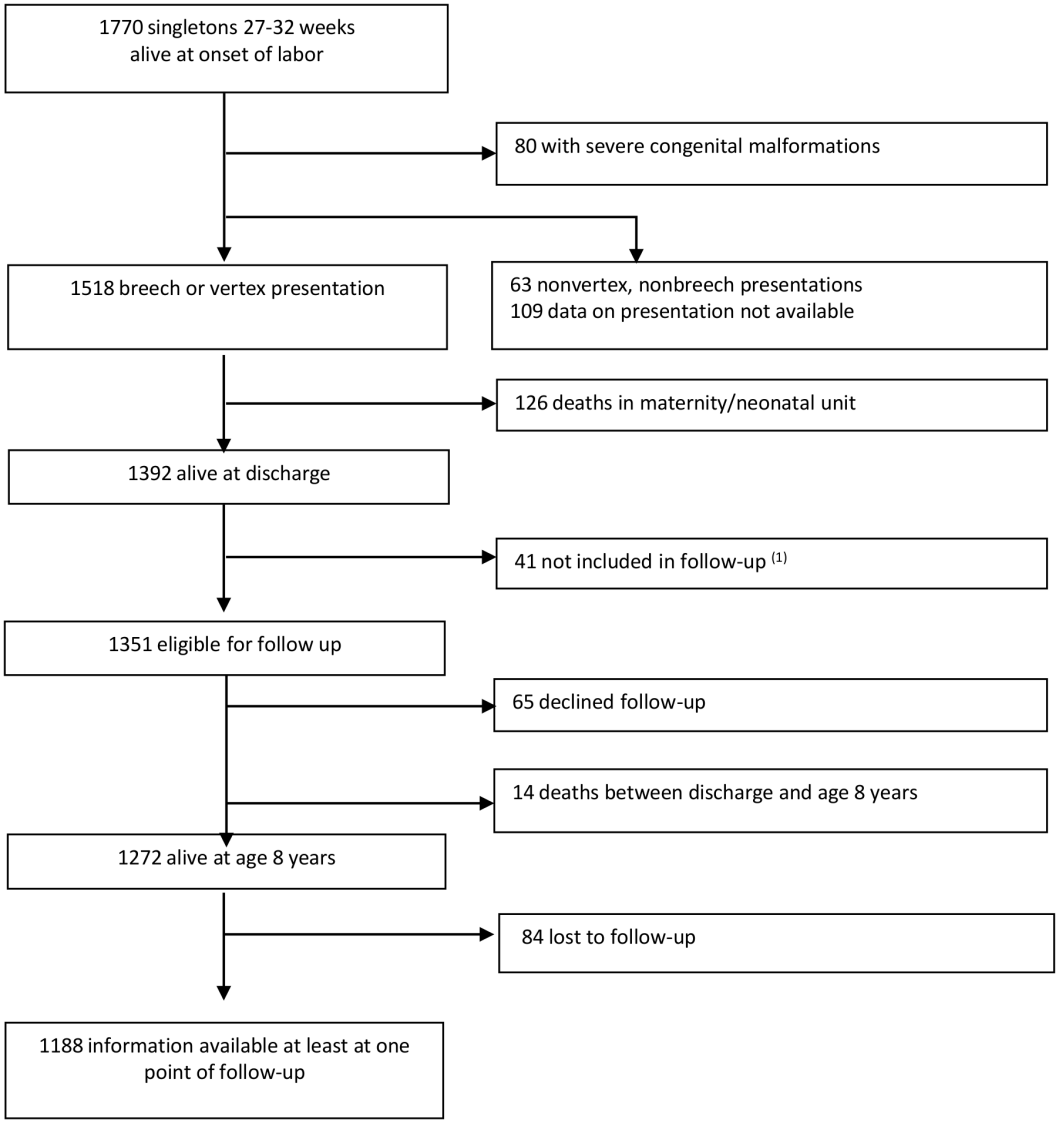
Study group. This figure is a flow chart describing the members of the EPIPAGE cohort included in this study. (1) In two regions, the protocol planned to include only half of the children born at 32 weeks in the follow-up.

After exclusion of 80 infants with severe congenital malformations (4.0% in the group of vertex presentation versus 5.4% in the group of breech presentation, *P* = 0.23) and infants with non-vertex and non-breech (n = 63) or unknown presentations (n = 109), this analysis included 1518 singleton infants alive at the beginning of preterm labor or before a cesarean delivery performed before labor at 27 to 32 weeks of gestational age (Fig 1). Among them, 1392 were alive at discharge; 41 were not included in the follow-up, i.e., half the infants born at 32 weeks in two regions, according to the initial protocol,[14] 65 families refused to participate in the follow-up, 14 children died between hospital discharge and the age of 8 years, and 84 were completely lost to follow-up (Fig 1). Finally, of the 1337 (1351–14) survivors at the age of 8 years, information on deficiencies was available for 1188 children (89%), the last point of follow-up being the age of 2 years for 98 children, the age of 5 years for 249 and, and the age of 8 for 841.

Mothers of the 149 (65+84) children lost to follow-up were more frequently younger than 25 years, multiparous, and more often had a lower SES, lower educational level, and a nationality other than French (data not shown). Presentation, antenatal corticosteroid administration, mode of delivery, gestational age at delivery, infant sex, and neonatal complications did not differ according to follow-up. However, among those born at 27–28 weeks of gestation, there was a trend (not statistically significant) for more lost to follow-up among those born in vertex presentation than among those born in breech presentation.

Of the 1518 fetuses alive before delivery with known presentation and without any severe congenital malformation, 351 (23.1%) were in breech presentation. Mothers of infants in breech presentation were significantly older than those of infants in vertex presentation. The groups did not differ for social characteristics (Table 2).

**Table 2:** Maternal and pregnancy characteristics.

	Vertex		Breech		
	n	%	n	%	p
**N**	1167		351		
**Maternal age at birth (years)**					
<25	334	28.8	74	21.3	0.01
25–34	660	56.9	211	60.6	
≥35	166	14.3	63	18.1	
**Parents’ socioeconomic status**^(1)^					
Professional	115	10.4	45	14.1	0.42
Intermediate	240	21.7	73	22.8	
Administrative, public service, self-employed, students	255	23.1	69	21.6	
Sales workers, service workers	191	17.3	52	16.3	
Manual workers, unemployed	303	27.4	81	25.3	
**Mother’s educational level**					
No school or primary school only	78	7.5	24	7.7	0.78
Middle school	490	46.9	145	46.8	
High school	206	19.7	68	21.9	
University	270	25.9	73	23.5	
**Country of birth**					
Other than France	162	15.1	52	16.2	0.65
France	908	84.9	269	83.8	
**Parity**					
0	588	50.5	157	44.7	0.08
1	431	37.0	153	43.6	
≥2	145	12.5	41	11.7	
**Previous cesarean section**					
No	1040	89.1	306	87.2	0.32
Yes	127	10.9	45	12.8	
**Complication of pregnancy**					
Maternal hypertension or SGA^(2)^	380	32.6	96	27.4	0.10
Spontaneous preterm labor or PPROM^(3)^, without hypertension or SGA	663	56.8	208	59.3	
Placenta previa, placental abruption, or other hemorrhage	60	5.1	28	8.0	
Other	64	5.5	19	5.4	

^(1)^ SGA: small for gestational age (defined as birth weight ≤10^th^ percentile—EPIPAGE internal reference)–SGA were by definition excluded from the sub group spontaneous preterm or PPROM

^(2)^ EPIPAGE internal reference

^(3)^ PPROM: Preterm premature rupture of membranes

Complications of pregnancy leading to the preterm birth were slightly but not significantly different, with fewer vascular complications among the breech presentations (Table 2). Overall the most common context of preterm delivery was spontaneous onset of labor or PPROM (n = 871, 57.4%). Breech deliveries occurred more frequently in level 3 maternity units, more often by cesarean section, and at earlier gestational ages. The same differences in mode of delivery and gestational age were observed when the analysis was restricted to the subset of mothers with spontaneous preterm labor or PPROM only (Table 3).

**Table 3:** Obstetric context according to fetal presentation.

	Total					Spontaneous preterm labor or PPROM				
	Vertex		Breech			Vertex		Breech		
	n	%	n	%	p	n	%	n	%	p
**N**	1167	*76*.*9*	351	*23*.*1*		663	*76*.*1*	208	*23*.*9*	
**Level of maternity unit**										
1	237	20.3	52	14.8	0.01	150	22.6	36	17.3	0.25
2	255	21.9	66	18.8		142	21.4	50	24	
3	675	57.8	233	66.4		371	56	122	58.7	
**Antenatal corticosteroids**										
No	338	29.7	84	24.6	0.07	203	31.3	62	30.4	0.80
Yes	801	70.3	257	75.4		445	68.7	142	69.6	
**Mode of delivery**										
Cesarean section before labor	121	10.4	87	25.1	<0.001	103	15.6	80	38.8	<0.001
Cesarean section during labor	508	43.6	171	49.3		43	6.5	43	20.9	
Vaginal delivery	537	46.1	89	25.6		516	77.9	83	40.3	
**Gestational age at delivery (weeks)**										
27–28	200	17.1	98	27.9	<0.001	123	18.6	63	30.3	<0.001
29–30	329	28.2	101	28.8		198	29.9	56	26.9	
31–32	638	54.7	152	43.3		342	51.6	89	42.8	
**Sex**										
Male	640	54.8	171	48.9	0.05	377	56.9	109	52.4	0.26
Female	527	45.2	179	51.1		286	43.1	99	47.6	
**SGA**^(1)^										
No	1059	90.7	314	89.7	0.56					
Yes	108	9.3	36	10.3						
**Birth weight**^(2)^										
<10^th^ percentile	108	9.3	36	10.3	0.33	0	0	0	0	0.90
[10-20^th^ [percentile	103	8.8	39	11.1		18	2.7	6	2.9	
≥ 20^th^ percentile	956	81.9	275	78.6		645	97.3	202	97.1	

^(1)^ SGA: small for gestational age (defined as birth weight ≤10^th^ percentile—EPIPAGE internal reference)–SGA were by definition excluded from the sub group spontaneous preterm or PPROM.

^(2)^ EPIPAGE internal reference.

Overall, neonatal mortality was significantly higher among infants in breech than vertex presentation (10.8% vs. 7.5%, *P* = 0.05). The difference was larger in the subset of infants born after spontaneous preterm labor or PPROM (13.0% vs.7.1%, *P* = 0.008) (Table 4).

**Table 4:** Mortality, neonatal morbidity, and long term neurodevelopmental outcome according to fetal presentation.

	Total					Spontaneous preterm labor or PPROM				
	Vertex		Breech			Vertex		Breech		
	n	%	n	%	p	n	%	n	%	p
**N**	1167		351			663		208		
**Neonatal mortality**										
No	1079	92.5	313	89.2	0.05	616	92.9	181	87.0	0.008
Yes	88	7.5	38	10.8		47	7.1	27	13.0	
**Mortality between neonatal unit discharge and age of 8 years**										
No	1050	99.0	307	99.0	0.92	599	98.4	177	98.3	0.98
Yes	11	1.0	3	1.0		10	1.6	3	1.7	
**Early-onset sepsis** ^(1)^										
No	1038	93.1	313	93.4	0.83	560	88.9	177	89.8	0.71
Yes	77	6.9	22	6.6		70	11.1	20	10.2	
**Late-onset neonatal infection** ^(2)^										
No	825	73.4	236	70.9	0.36	498	77.6	146	74.5	0.37
Yes	299	26.6	97	29.1		144	22.4	50	25.5	
**Necrotizing enterocolitis**										
No	1084	96.7	328	97.6	0.39	619	96.7	194	98.0	0.36
Yes	37	3.3	8	2.4		21	3.3	4	2.0	
**Bronchopulmonary dysplasia** ^(3)^										
No	1008	91.2	289	88.4	0.12	579	92.3	176	89.8	0.26
Yes	97	8.8	38	11.6		48	7.7	20	10.2	
**Neonatal cerebral lesion** ^(4)^										
Major	69	6.3	19	5.8	0.91	47	7.5	16	8.3	0.92
Moderate	138	12.5	45	13.7		79	12.6	26	13.5	
Minor	158	14.3	44	13.4		88	14	24	12.5	
None	737	66.9	220	67.1		413	65.9	126	65.6	
**Neuromotor deficiencies** ^(5)^										
Severe CP ^(6)^	36	3.9	2	0.7	0.03	33	6.3	2	1.2	0.09
Moderate CP	52	5.4	15	5.6		32	5.9	12	7.9	
No CP, other motor disorder	33	3.4	15	5.6		20	3.7	5	3.7	
None identified	791	87.3	244	88.2		434	84.1	137	87.2	
**Cognitive deficiencies/learning disabilities** ^(5)^										
Severe	53	5.9	24	8.7	0.20	29	5.5	16	9.8	0.16
Moderate	212	22.9	56	20.2		120	22.7	32	20.1	
None identified	647	71.3	196	71.1		370	71.8	108	70.1	
**Overall deficiencies** ^(5)^										
Severe	97	10.7	29	10.5	0.81	60	11.4	19	11.6	0.99
Moderate	251	27.0	69	25.1		134	25.3	39	25.0	
None identified	564	62.4	178	64.5		325	63.3	98	63.4	

^(1)^ Defined as confirmed infection of maternal origin (vertically transmitted).

^(2)^ Defined as a postnatally acquired infection (horizontally acquired) treated with antibiotics for at least 7 days.

^(3)^ 0xygen at 36 weeks.

^(4)^ Cerebral lesions included major lesions (cystic periventricular leukomalacia or intraparenchymal hemorrhage), moderate lesions (intraventricular hemorrhage (IVH) with primary ventricular dilation or ventricular dilatation or persistent echodensity), minor lesions (subependymal hemorrhage or IVH without ventricular dilation) and no lesion.

^(5)^ See also Table 1.

^(6)^ CP: cerebral palsy.

The differences were no longer significant, however, after we controlled for gestational age and other potential confounders (Table 5). Mortality after discharge was low (1%) and did not differ according to fetal presentation (Table 4). The groups did not differ significantly for neonatal morbidity (Table 4). Severe cerebral palsy seemed to be less frequent in the breech group (Table 4), but there was no significant difference after adjustment (Table 5). There was no difference according to presentation for cognitive deficiencies/learning disabilities or overall deficiencies (Tables 4–5).

**Table 5:** Neonatal mortality and long term neurodevelopmental outcome according to fetal presentation, multivariate analyses.

	Total			Spontaneous preterm labor or PPROM		
	Breech vs vertex			Breech vs vertex		
	N	OR (95%CI)		N	OR (95%CI)	
Neonatal mortality						
Crude OR	1518	1.49	1.00–2.22	871	1.96	1.18–3.23
AdjOR1	1480	1.12	0.71–1.76	852	1.49	0.87–2.58
AdjOR2	1472	1.15	0.73–1.82	852	1.53	0.89–2.65
AdjOR3	1469	1.09	0.68–1.73	849	1.40	0.78–2.49
Neuromotor deficiencies^(1)^						
Severe or moderate CP						
Crude OR	1188	0.65	0.38–1.13	675	0.73	0.39–1.35
AdjOR1	1168	0.60	0.34–1.05	663	0.68	0.36–1.29
AdjOR2	1162	0.58	0.33–1.02	663	0.68	0.36–1.30
AdjOR3	1160	0.67	0.37–1.21	661	0.81	0.41–1.61
Cognitive deficiencies / learning disabilities ^(1)^						
Severe or moderate						
Crude OR	1188	1.01	0.75–1.37	675	1.08	0.73–1.61
AdjOR1	1168	0.95	0.70–1.30	663	1.05	0.70–1.56
AdjOR2	1162	0.96	0.70–1.31	663	1.06	0.71–1.58
AdjOR4	1130	0.86	0.61–1.23	642	0.96	0.60–1.55
Overall deficiencies^(1)^						
Severe or moderate						
Crude OR	1188	0.91	0.69–1.22	675	0.99	0.68–1.45
AdjOR1	1168	0.86	0.64–1.16	663	0.96	0.65–1.41
AdjOR2	1162	0.88	0.65–1.18	663	0.99	0.67–1.45
AdjOR4	1130	0.85	0.62–1.17	642	1.02	0.66–1.58

adjOR1: adjusted for gestational age, sex, and antenatal corticosteroids. adjOR2: adjusted for gestational age, sex, antenatal corticosteroids, SGA, and PPROM. adjOR3: adjusted for gestational age, sex, antenatal corticosteroids, SGA, PPROM, maternity unit level, and mode of delivery. adjOR4: adjusted for gestational age, sex, antenatal corticosteroids, SGA, PPROM, maternity unit level, mode of delivery, parental socioeconomic status, maternal age at delivery, and country of birth.

^(1)^ See Table 1

## Discussion

### Main findings

The main finding of this study is that in very preterm deliveries, breech presentation, compared with vertex, was not an independent risk factor for neonatal mortality or long-term neurological deficiencies. Before adjustment, a difference in mortality according to presentation was observed, especially when the analysis was restricted to infants delivered after spontaneous preterm labor or PPROM. However, as previously observed by Goodman et al in a retrospective study designed to assess differences in outcomes between vertex and nonvertex presentations in case of PPROM between 24 and 34 weeks’ gestation [17], gestational age at delivery in EPIPAGE was lower in the breech group. This difference in gestational age is likely to explain both the higher crude neonatal mortality rate of breech infants in both studies and the disappearance of this difference after adjustment for gestational age in our study.

The increased risk of severe CP in the group of infants in vertex presentation compared to breech is of concern. However our study is a secondary analysis of Epipage data, and the cohort was not designed to compare the incidence of cerebral palsy between breech and vertex fetus. Numbers were small, especially for breech presentations. When severe and moderate CP were considered together, there was no significant difference according to fetal presentation. For both outcomes, we cannot completely rule out the possibility that the absence of statistical significance for a small difference after adjustment may result from a lack of power. But the study had a power of 98% to detect a risk of cerebral palsy multiplied by 2 and of 50% to detect a risk of cerebral palsy multiplied by 1.5.

### Strengths and limitations

Besides a long-term follow-up and the assessment of outcomes at 2, 5, and 8 years (school age), the strengths of the EPIPAGE study are its prospective design, its geographical basis, and its large sample size: 9 regions covering one third of the annual births in the country. As evidenced by the rarity of publications on whether breech presentation is an independent risk factor for adverse outcome among preterm infants, the question is difficult to address, especially because the causes of the preterm delivery might well mask perinatal and long-term mortality and morbidity related to the presentation. It thus requires large cohorts of infants born preterm with known details about the immediate reason for preterm delivery and mode of delivery. It also requires long-term follow-up of these preterm infants, as before school age, subtle deficiencies leading to learning difficulties remain undetected. This study fulfills both requirements.

Although born in 1997, our cohort is nonetheless more recent than most other studies of this question and thus more likely to reflect current obstetric and neonatal care practices. Despite the changes since then, in-utero transfer, antenatal steroids, early surfactant administration, and new ventilation techniques were widely used in France in 1997. While more recent data would have been valuable, studies such as EPIPAGE 2 of preterm deliveries in 2011 [27] would not allow information on outcome at school age before several years.

A limitation inherent to long-term cohort studies is the attrition bias: some families moved and others never replied even though they did not explicitly decline to participate. This phenomenon affects our cohort, but information about deficiencies is available for at least one follow-up point for 89% of the children. This follow-up rate should be considered in light of the large number of children included, the substantial geographical dispersion, and the mobility of parents with young children. Nonetheless, this loss to follow-up can be associated with the underestimation of unfavorable outcomes. This bias has been observed in previous studies, where the children lost to follow-up had a higher rate of cerebral lesions at neonatal ultrasound scans and a lower SES [28]. In our study, the SES of families of children lost to follow-up was lower than for those who continued to participate, but rates of neonatal cerebral lesions were similar. The fact that children lost to follow-up were more likely to belong to families with a lower SES raises concerns about the potential underestimation of cognitive deficiencies [29]. Furthermore, we were not able to assess the cognitive performance of the 98 children followed only until the age of 2. However the proportion of children lost to follow up did not vary according to presentation, and it is unlikely that it biased the comparison.

### Interpretation

Most studies of preterm breech have focused on mode of delivery, and very few have been designed to determine whether fetal presentation is an independent risk factor for adverse outcomes [11,12]. Demol et al. did focus on neonatal mortality in preterm infants, comparing 692 non-vertex to 4685 vertex infants born between 1985 and 1995 [12]. In contrast with our results, they reached the conclusion that breech presentation in preterm delivery is an independent risk factor for neonatal mortality (adjOR = 2.2 95%CI 1.37–3.57). No follow-up data were available. Several factors may explain this difference with our results; (i) although the gestational age of the infants included in their analysis ranged from 24 to 36 weeks, 79.6% of their study population had a gestational age of 33 to 36 weeks and 5.6% between 24–27 weeks; they thus differ quite substantially from our population aged 27–32 weeks of gestation; (ii) their study was retrospective, whereas ours was prospective; (iii) severe congenital anomalies, significantly more frequent in both nonvertex groups, were not excluded from their analysis but were in ours. Our decision to exclude these infants was made for two reasons. First, previous reports indicated that an increased frequency of such anomalies could be expected in nonvertex presentations. Although not significant, the same trend was observed in our cohort. Second, a known congenital anomaly is likely to lead clinicians to approach management of delivery and/or neonatal care with a somewhat different attitude.

Like us, Gravenhorst et al. also excluded congenital anomalies in their report of the Dutch nation-wide study of very preterm or very low birth weight infants born in 1983 (POPS),[11] and they observed an increased risk of neonatal mortality for breech compared to vertex (adjOR = 1.6, *P*<0.05). However the inclusion of infants of very low birth weight of higher gestational ages, may modify the results. At the age of 5, the adjusted risk of handicap in the two groups did not differ.

## Conclusion

In conclusion, our data suggest that breech presentation is not an independent risk factor for neonatal mortality or long-term neuromotor or cognitive deficiencies for very preterm infants. Our results can help physicians provide useful information to parents.
